# Stakeholder Engagement to Identify Priorities for Improving the Quality and Value of Critical Care

**DOI:** 10.1371/journal.pone.0140141

**Published:** 2015-10-22

**Authors:** Henry T. Stelfox, Daniel J. Niven, Fiona M. Clement, Sean M. Bagshaw, Deborah J. Cook, Emily McKenzie, Melissa L. Potestio, Christopher J. Doig, Barbara O’Neill, David Zygun, Critical Care Strategic Clinical Network, Alberta Health Services

**Affiliations:** 1 Department of Critical Care Medicine, University of Calgary, Calgary, Alberta, Canada; 2 Department of Community Health Sciences, University of Calgary, Calgary, Alberta, Canada; 3 Alberta Health Services, Alberta, Canada; 4 Division of Critical Care Medicine, Faculty of Medicine and Dentistry, University of Alberta, Edmonton, Alberta, Canada; 5 Departments of Medicine, Critical Care, and Clinical Epidemiology and Biostatistics, McMaster University, and St. Joseph's Healthcare, Hamilton, Ontario, Canada; 6 O’Brien Institute of Public Health, University of Calgary, Calgary, Alberta, Canada; Centre Hospitalier Universitaire Vaudois, FRANCE

## Abstract

**Background:**

Large amounts of scientific evidence are generated, but not implemented into patient care (the ‘knowledge-to-care’ gap). We identified and prioritized knowledge-to-care gaps in critical care as opportunities to improve the quality and value of healthcare.

**Methods:**

We used a multi-method community-based participatory research approach to engage a Network of all adult (n = 14) and pediatric (n = 2) medical-surgical intensive care units (ICUs) in a fully integrated geographically defined healthcare system serving 4 million residents. Participants included Network oversight committee members (n = 38) and frontline providers (n = 1,790). Network committee members used a modified RAND/University of California Appropriateness Methodology, to serially propose, rate (validated 9 point scale) and revise potential knowledge-to-care gaps as priorities for improvement. The priorities were sent to frontline providers for evaluation. Results were relayed back to all frontline providers for feedback.

**Results:**

Initially, 68 knowledge-to-care gaps were proposed, rated and revised by the committee (n = 32 participants) over 3 rounds of review and resulted in 13 proposed priorities for improvement. Then, 1,103 providers (62% response rate) evaluated the priorities, and rated 9 as ‘necessary’ (median score 7–9). Several factors were associated with rating priorities as necessary in multivariable logistic regression, related to the provider (experience, teaching status of ICU) and topic (strength of supporting evidence, potential to benefit the patient, potential to improve patient/family experience, potential to decrease costs).

**Conclusions:**

A community-based participatory research approach engaged a diverse group of stakeholders to identify 9 priorities for improving the quality and value of critical care. The approach was time and cost efficient and could serve as a model to prioritize areas for research quality improvement across other settings.

## Introduction

Large amounts of scientific evidence are generated, yet not effectively implemented into patient care (the ‘knowledge-to-care’ gap).[1] Healthcare systems routinely fail to make optimal use of evidence, which results in suboptimal, low value patient care (i.e., underuse, overuse and misuse of therapies).[2] Identifying and addressing knowledge-to-care gaps is an opportunity to improve the quality and value of healthcare by implementing high value patient care practices.[3]

The best approach to identify and prioritize knowledge-to-care gaps for intervention is unknown.[4] Historically, priorities have been independently established by individuals and organizations engaged in research and quality improvement.[5] However, because of growing concerns about the relevance of research and quality improvement to human health, healthcare systems are increasingly looking to engage stakeholders in establishing priorities.[5] Employing research-practice partnerships holds the promise of better matching stakeholders’ needs with research and quality improvement efforts, while engaging stakeholders to lead and sustain practice change.[6]

In 2013, Alberta Health Services, a fully integrated geographically defined healthcare system serving a population of 4 million residents, launched a program with Alberta Innovates Health Solutions (research funder) to improve value for money in healthcare by encouraging de-adoption of low value patient care practices and adoption of high value patient care practices.[7] In response, a network (Critical Care Strategic Clinical Network)[8] of all adult (n = 14) and pediatric (n = 2) medical-surgical intensive care units (ICUs) in the healthcare system conducted a research project to evaluate the feasibility of using a research-practice partnership to identify and prioritize knowledge-to-care gaps in the care of critically ill patients admitted to ICU as opportunities to improve the quality and value of healthcare.

## Methods

### Study Design and Population

Identifying and addressing knowledge-to-care gaps requires a coordinated multi-step longitudinal process that includes identifying problems, developing and implementing locally tailored interventions, evaluating the outcomes and monitoring the sustainability of the practice change.[9] The complexity and applied nature of the problem requires a research-practice partnership.[10] We selected a community-based participatory research approach where researchers and ICU stakeholders are equitably partnered, because it balances scientific rigor with community engagement so that practice change can be informed by science, but implemented, scaled and sustained by stakeholders.[6, 11] We used the ‘knowledge-to-action’ (KTA) cycle developed by Graham et al.[9] as the guiding framework for the study given its review of more than 30 theories of planned action and its successful use in similar clinical contexts.[12, 13]

We conducted a multi-method study to identify and prioritize common patient care practices (e.g., tests, treatments, processes of care) that may be underused, overused or misused in medical-surgical ICUs. The goal was to inform a subsequent audit of patient care, as part of the Critical Care Strategic Clinical Network’s program to systematically de-adopt low value patient care practices and adopt high value patient care practices. First, we used a modified RAND/University of California appropriateness method, a valid and reliable deliberative process, to identify common patient care practices with perceived knowledge-to-care gaps as priorities for improvement.[14, 15] Second, we surveyed frontline providers to evaluate the proposed priorities for improvement.[16] Third, we relayed the results back to frontline providers for feedback and to ascertain their engagement to participate in resulting quality improvement initiatives. We defined a knowledge-to-care gap as a discrepancy between what current scientific evidence suggests is the best practice, and the actual care provided.[17] We defined ICU stakeholders as providers, managers and decision-makers responsible for patient care. To be inclusive, ICUs that serve both adult and pediatric patients were included.

### Identification of Priorities for Improvement

We invited members of the Critical Care Strategic Clinical Network oversight committee to participate in a modified RAND/University of California appropriateness method to identify common patient care practices with perceived knowledge-to-care gaps as priorities for improvement.[15] The committee is a standing body comprised of multidisciplinary providers, managers and decision-makers representing all ICUs in the healthcare system and meets monthly to provide oversight of the Network. Committee members were divided into two separate focus groups (March 20, 2014) and asked to consider three common types of knowledge-to-care gaps: (1) over use—when science shows a patient care practice is ineffective or harmful, but is prescribed (e.g., tight glycemic control in critically ill patients);[18, 19] (2) under use—when science shows a practice is effective, but is not prescribed (e.g., venous thromboembolism prophylaxis);[20, 21] and (3) misuse—when science shows a practice to be effective, but is prescribed for the wrong patients, wrong reason or wrong time (e.g., prescription of albumin in patients with traumatic brain injury, but not in patients with cirrhosis and spontaneous bacterial peritonitis)[22, 23] (S1 Appendix–focus group guide). We included suggestions spanning the spectrum of scientific evidence (i.e., quality of evidence did not serve as an inclusion or exclusion criterion). In addition, participants were asked what criteria should be considered when evaluating patient care practices as potential priorities for improvement. Suggestions were recorded by a note taker, reviewed with the participants and collated. Audiotaped recordings of the proceedings were reviewed to ensure all suggestions were documented. Two members of the research team independently reviewed the lists of practices and criteria from the two focus groups, combined suggestions and refined terminology for consistency. Disagreements were resolved with discussion.

The rating process consisted of two rounds of sequential review and revision. Committee members were presented with a list of the priorities and asked to independently rate the priorities according to their importance as opportunities for improving the quality and value of healthcare using the validated nine-point RAND/University of California appropriateness method scale.[15] Committee members were asked to provide written comments. Round one was performed using a secure, web-based survey. Round two was performed using a half-day in-person workshop (May 15, 2014, Edmonton, Canada). Committee members were provided with personalized rating summaries from round one (i.e., individual priority ratings assigned by the committee member, anonymous distribution of ratings by other committee members, summary of committee member comments). Each priority was presented by a moderator, discussed and independently rerated by committee members.

### Evaluation of Priorities for Improvement

We surveyed all frontline providers working in adult (n = 14 ICUs) and pediatric (n = 2 ICUs) medical-surgical (medical, surgical, trauma, transplant) ICUs in Alberta, Canada. Providers were identified using login identifications for electronic medical record systems and patient care manager staff lists. We sent an email cover letter explaining the purpose of the study and a link to a secure web-based survey. The survey included an evaluation framework comprised of six criteria proposed by committee members. Members of the research team, working independently in groups of two, produced a synthesis of the criteria for each priority using a modified Grades of Recommendation Assessment, Development, and Evaluation (GRADE) system: strength of supporting evidence (searched literature), potential to benefit the patient (searched literature), potential to improve patient/family experience (solicited perspectives of patient/family network advisors), potential to decrease costs (reviewed costing data), ability to easily measure (reviewed electronic medical record data elements), and ability to act upon (solicited perspectives of network decision-makers).[24] We asked participants to rate the priorities according to their importance as opportunities for improving the quality and value of healthcare using the same validated nine-point scale and to suggest additional priorities. Participation in the study was voluntary and anonymous; written consent was not requested, but inferred with survey completion. Reminders were sent weekly (survey launched June 4, 2014 and closed July 2, 2014). Participation was encouraged by widely advertising the survey, recruiting local champions to promote participation and offering weekly draws for coffee cards.[16]

### Community Engagement

The results of the provider surveys were collated and relayed back to all providers (September 17, 2014). An attached survey asked providers to indicate whether the priorities rated as necessary were reasonable choices for quality improvement initiatives, whether they were supportive of working on future initiatives targeting the priorities, and whether they would be willing to act as a local champion for the initiatives. We provided no reminders as the primary purpose of the communication was to relay results back to providers (survey closed October 2, 2014) and we wanted to minimize participant burden given the programmatic nature of the work.

### Statistical Analysis

The primary analysis was to describe priority ratings by committee members and providers. The median rating was used to classify each priority as unnecessary (median score 1–3), supplementary (median score 4–6) or necessary (median score 7–9). Priorities rated as necessary by committee members and providers were considered potential priorities for improvement. We tested for associations between provider and priority characteristics, and the ratings provided by providers, using mixed effects logistic regression with clustering by respondent (i.e., to account for the rating of multiple priorities by each respondent).[25–28] The unit of analysis was each respondent’s rating of each priority (responses dichotomized necessary vs. unnecessary/supplementary). We evaluated for potential interactions between significant provider and priority characteristics. All analyses were conducted using Stata version 13.1 (Stata Corp, College Station, TX), with a *p*-value < 0.05 defining statistical significance. Ethics approval was obtained from the Conjoint Health Research Ethics Board at the University of Calgary.

## Results

### Identification of Priorities for Improvement

**Fig 1:**
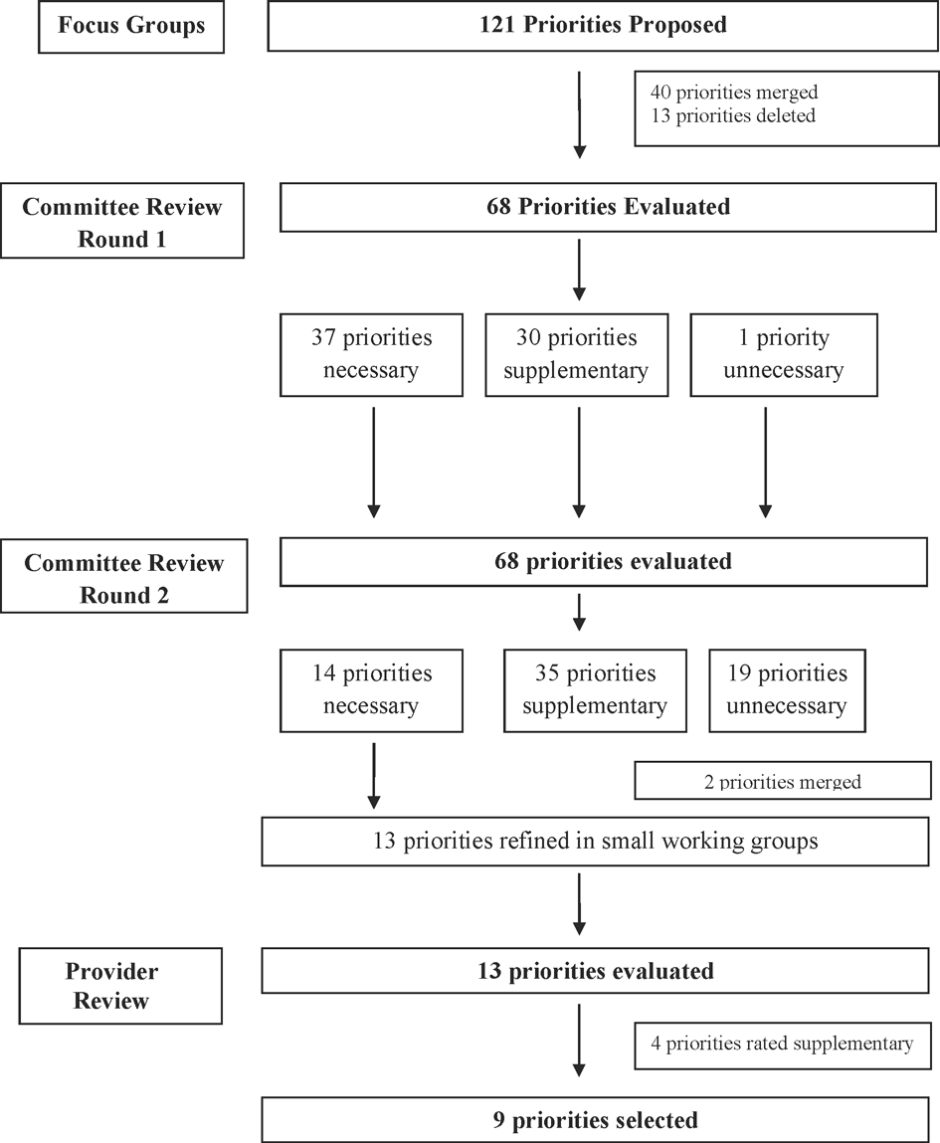
Flow Chart of Priorities for Improvement.

Of the 38 network committee members, 32 (84%) participated in the consensus process (Table 1). Fig 1 summarizes the flow of priorities for improvement through the study. Committee members proposed 121 priorities between two focus groups. After combining 40 duplicates and deleting 13 priorities that did not satisfy inclusion criteria (e.g., not a direct patient care practice) a total of 68 priorities were proposed. Committee members were presented with all 68 priorities at the beginning of the review process (S2 Appendix). Two priorities, strategies to facilitate patient day/night cycle and strategies to preserve patient sleep, were merged. Nineteen priorities were rated as unnecessary. Table 2 summarizes the 13 priorities for improvement rated as necessary.

**Table 1:** Characteristics of Critical Care Network Committee Members and Providers.* ^†^

Characteristics	Committee Members N = 32	Providers N = 1,103
Profession		
Physician	15 (47)	78 (7)
Nurse	14 (44)	676 (61)
Respiratory therapist	2 (6)	200 (18)
Allied health^‡^	1 (3)	149 (13)
Primary role		
Direct patient care	18 (56)	1,029 (93)
Administration	24 (75)	105 (9)
Teaching providers	14 (44)	224 (20)
Research	3 (9)	65 (6)
Other	2 (6)	38 (3)
Years of experience in critical care, median (IQR)	18 (10–22)	7 (3–14)
Academic status of ICU^§^		
Non-teaching	14 (44)	233 (21)
Teaching	24 (75)	845 (77)
Types of patients cared for^¶^		
Adult	28 (87)	967 (88)
Pediatric	4 (12)	134 (12)

* Unless otherwise indicated, data are expressed as number (%).

^†^ Counts may sum to greater than 100% as participants reported more than response for some characteristics

‡Allied health includes pharmacists, physiotherapists and social workers.

^§^Data missing for 25 providers

^¶^Data missing for 2 providers.

**Table 2:** Summary of Critical Care Network Committee and Provider Ratings.*

Priorities	Median Score on 9 Point Scale^†^ (IQR)	
	Committee Ratings	Provider Ratings
End-of-life care	7 (6, 7)	8 (7, 9)
Early mobilization	8 (6, 9)	8 (7, 9)
Strategies to preserve patient sleep	7 (6, 7)	8 (7, 8)
Establishing daily goals for patient care	7 (5, 8)	7 (6, 8)
Transition of patient care from ICU to hospital ward	8 (6, 9)	7 (6, 8)
Transition of patient care between providers within ICU	7 (6, 8)	7 (5, 8)
Daily sedation interruption	7 (5, 8)	7 (5, 8)
Delirium screening & diagnosis	7 (7, 9)	7 (5, 8)
Temperature control in patients after resuscitation from cardiac arrest	7 (3, 7)	7 (5, 8)
Duration of empiric antimicrobial prescriptions	7 (5, 8)	6 (5, 8)
Physical and pharmacological restraints	7 (4, 7)	6 (5, 7)
Patient and family participation in daily rounds	7 (5, 7)	6 (5, 8)
Routine blood tests	7 (5, 8)	6 (5, 7)

* Data presented for the 13 priorities for improvement rated as necessary by network committee members

Abbreviations: IQR, interquartile range

^†^ The median rating was used to classify each priority as unnecessary (median score 1–3), supplementary (median score 4–6) or necessary (median score 7–9).

Committee members proposed six criteria to guide selection of priorities during the focus groups and rated the criteria according to importance during the first round of reviews. Committee members rated potential to benefit patient (median score 9, interquartile range [IQR] 8–9), ability to easily measure the practice (median score 8, IQR 7–9), ability to take action to change the practice (median score 8, IQR 7–9), strength of supporting evidence (median score 8, IQR 7–9), and potential to improve patient/family experience (median score 7, IQR 6–8) as necessary criteria, and potential to decrease costs (median score 6, IQR 5–8) as a supplementary criterion.

### Evaluation of Priorities for Improvement

We surveyed 1,790 providers and received 1,103 responses (62% response rate) from providers who reported working in 16 medical-surgical ICUs in seven cities. The majority of respondents were nurses (61%) followed by respiratory therapists (18%), allied health professionals (13%) and physicians (7%) (Table 1). Most provided direct patient care (93%) to adult patients (88%) in teaching (77%) ICUs. Respondents worked a median of 7 years in critical care.

Table 2 summarizes provider ratings (S3 Appendix). Of the 13 priorities for improvement rated as necessary by network committee members, nine were rated as necessary by providers (i.e., median score 7–9): end-of-life care, early mobilization, strategies to preserve patient sleep, transition of patient care from ICU to hospital ward, transition of patient care between providers within the ICU, daily sedation interruption, delirium screening & diagnosis, and temperature control in patients after resuscitation from cardiac arrest. One hundred and thirty six providers proposed 55 additional unique priorities for improvement, of which 19 had not been previously considered by the network committee (S4 Appendix).

Ratings were consistent across provider and institution characteristics with a few notable exceptions (S5 Appendix). Among the priorities rated as supplementary, nurses and respiratory therapists rated the use of physical and pharmacological restraints as necessary, physicians rated the duration of empiric antimicrobial prescriptions as necessary and allied health providers rated patient and family participation in daily rounds as necessary.

### Characteristics of the Priorities for Improvement

Table 3 summarizes the multivariable adjusted odds ratios of provider and priority characteristics associated with provider ratings. The odds of physicians, nurses and respiratory therapists rating a priority as necessary were not significantly different. Provider years of experience were associated with an increased likelihood of priorities being rated as necessary. Providers working in non-teaching ICUs were 20 percent (95% confidence interval, 3% to 40%) more likely to rate priorities as necessary than providers working in teaching ICUs. The characteristics of the priorities associated with those rated as necessary included strength of supporting evidence, potential to benefit the patient, potential to improve patient/family experience, and potential to decrease costs. The perceived ability to easily measure the practice was not associated with priorities rated as necessary. The perceived ability to take action to change the practice was inversely associated with priorities rated as necessary. There was a significant interaction between respondent profession and the potential to decrease costs (*p* < 0.001)(S6 Appendix). For priorities with a neutral effect on costs, nurses, respiratory therapists, and allied health practitioners were more likely than physicians to rate the priority as necessary. For priorities with potential to decrease costs, there was no difference across respondent professions.

**Table 3:** Characteristics Associated with Priorities Rated as Necessary by Providers.

Characteristic	Adjusted Odds Ratio (95% confidence interval)*	P-value*
Provider Characteristics		
Profession		0.001
Physician^†^	1.0	
Nurse	1.07 (0.83–1.36)	
Respiratory Therapist	1.08 (0.82–1.42)	
Allied Health	1.57 (1.17–2.11)	
Years of experience in critical care		<0.001
Less than 10 years^†^	1.0	
10 to 20 years	1.24 (1.08, 1.43)	
More than 20 years	2.02 (1.66, 2.47)	
Academic status of ICU		
Teaching^†^	1.0	
Non-teaching	1.20 (1.03, 1.40)	0.016
Priority Characteristics		
Strength of supporting evidence	2.70 (2.48–2.95)	<0.001
Potential to improve patient/family experience	1.51 (1.34–1.71)	<0.001
Potential to benefit the patient	1.61 (1.45–1.80)	<0.001
Potential to decrease costs	1.25 (1.12–1.39)	<0.001
Ability to easily measure the practice	1.06 (0.92–1.22)	0.398
Ability to take action to change the practice	0.90 (0.82–0.99)	0.032

* Odds ratios and p-values adjusted for characteristics listed in table using random effects logistic regression models. Odds ratios greater than one indicate increased odds of being rated as necessary by Providers. Odds ratios less than one indicate decreased odds of being rated as necessary by providers.

^†^ Patients with this factor served as the reference group.

### Community Engagement

The results of the provider survey were relayed back to providers. A total of 627 providers responded (35% response rate) and indicated that the nine priorities rated as necessary were reasonable choices for quality improvement initiatives (87%) and that they were highly supportive of working on future initiatives targeting the priorities (61%). Ninety-two individuals representing nurses, respiratory therapists, physicians and allied health professionals working in adult, pediatric, teaching and non-teaching ICUs indicated that they would be willing to act as local champions for future initiatives targeting these priorities.

## Discussion

In response to a request from a healthcare system, we tested the feasibility of using a community-based participatory research approach to engage a diverse group of stakeholders to identify and prioritize opportunities to improve the quality and value of critical care. We trialed the use of technically robust techniques as part of the approach using a modification of the RAND/UCLA Appropriateness Methodology to identify priorities and survey of frontline providers to evaluate them. We proposed and tested six evaluation criteria that can be used to frame future prioritization activities. The approach, methods and evaluation criteria we employed can be used as a template for identifying priorities across other settings.

Different approaches have been used to identify priorities for research and quality improvement. Scientists have traditionally advocated the use of valid and reliable tools with robust evaluative methodologies (e.g., consensus methods), but have been criticized for moving too slowly and having ‘ivory tower’ perspectives.[29] Healthcare decision-makers have traditionally advocated efficient and nimble quality improvement strategies (e.g., Plan-Do-Study-Act cycles), but have been criticized for employing methodologies at risk of bias.[30] The strength of the research-practice partnership employed in our study is that it combines the strengths of these two approaches. For example, the Choosing Wisely^®^ initiative to identify and publicize low value tests or procedures has resulted in over 50 professional societies publishing top five lists and spread internationally to other healthcare systems.[31, 32] Such initiatives have been criticized for a lack of rigor and transparency in the methods employed in selecting priorities.[33, 34] Our methodology demonstrates the feasibility of using a community-based participatory research approach that is scientifically robust and engages all stakeholders involved in multidisciplinary patient care. This approach has been successfully used in public health, but infrequently in hospital-based healthcare.[11] For example, a search of the National Library of Medicine identified 18 articles indexed with the exploded Medical Subject Headings ‘hospitals’ and ‘community-based participatory research’. We were able to identify two articles within the clinical area of critical care, one review article[35] and one commentary[36], but no original research reports. In contrast, we identified over seventeen hundred articles of community-based participatory research in public health. Similar approaches have been adopted in non-healthcare industries to guide quality improvement efforts.[37]

Our research highlights a different conceptual approach to improving quality and value in healthcare. Traditionally, knowledge transfer activities have focused on facilitating the adoption of evidence informed technologies into practice.[17] Conversely, the Choosing Wisely^®^ initiative has focused on eliminating waste from practice.[31] We employed a modification of these two approaches, reasoning that closing knowledge-to-care gaps by reducing the underuse, overuse or misuse of patient care practices will improve the quality and value of care. The Choosing Wisely^®^ Top 5 List in Critical Care Medicine, nested within the priorities considered in our approach, highlights this nicely.[38] In addition to independently considering these items, providers in our project selected patient care practices perceived to be underused (e.g., early mobilization) or misused (e.g., transitions of patient care). Of the Choosing Wisely^®^ Top 5 List, two items were rated as necessary priorities for improving the quality and value of care by stakeholders within our critical care community (end-of-life care, daily sedation interruption), two as supplementary priorities (routine blood tests/diagnostic imaging studies, limiting blood transfusion in non-bleeding patients) and one as an unnecessary priority (providing total parenteral nutrition).[38] This highlights that priorities for improvement are likely to vary across jurisdictions and institutions, and underscores the importance of engaging local stakeholders to ensure initiatives match local needs.

We found that consideration needs to be given to both stakeholder characteristics and patient care practice characteristics when selecting priorities for improvement. Although stakeholders of different backgrounds demonstrate similar patterns of selection, those working in non-teaching hospitals were more likely to rate priorities for improvement as necessary than those working in teaching hospitals, suggesting a greater openness or perceived need for improvement. Providers selected priorities supported by scientific evidence and potential to benefit the patient/family. Surprisingly, the logistical practicalities of the ability to easily measure the practice and to take action to change the practice were not positively associated with priority selection. Cost only modestly influenced providers. These data reinforce previous observations that providers may be more motivated to improve care of their patients than to act as stewards of the healthcare system.[39, 40] Future efforts to improve value of healthcare may benefit from accompanying educational initiatives that highlight the importance of stewardship in improving and sustaining quality of care.[41]

Our study has both important strengths and limitations. First, our study reports perceived priorities for improvement, but does not document actual deficiencies in care. This is an important distinction because quality improvement initiatives have previously been initiated in response to stakeholder priorities only to discover that opportunities for improvement were smaller than anticipated.[42] An audit of patient care is required to document gaps as well as establish facilitators and barriers to practice change before designing and implementing interventions. Second, our project reports anonymous data from multiple institutions across a geographically defined healthcare system. Although the rating of priorities was similar across institutions with different characteristics (e.g., urban vs. regional, non-teaching vs. teaching, adult vs. pediatric), differences may exist between individual institutions and should be considered during healthcare system wide initiatives. Finally, our study does not provide any information about patient and family priorities, which may differ from those of providers, managers and decision-makers, and were solicited through a separate process[43]. Future work is needed to bring together the perspectives of the different stakeholders into a ‘reconciled list of provider, decision-maker, patient and family priorities’ to inform the resulting audit and improvement initiatives. The strengths of our study include its applied nature, population-based design and community-based participatory research approach.

## Conclusions

In summary, we successfully used a community-based participatory research approach to engage a diverse group of multidisciplinary stakeholders to identify and prioritize knowledge-to-care gaps in the care of critically ill patients. We tested the use of valid and reliable scientific methods as part of the approach. We proposed and tested six evaluation criteria that can be used to frame future prioritization activities. The nine priorities rated as necessary by stakeholders will inform initiatives to improve the quality and value of care. The approach, methods and evaluation criteria we employed can be used as a template for identifying priorities across other settings.

## Supporting Information

S1 Appendix Focus Group Guide. (PDF)

S2 Appendix Critical Care Network Core Committee Ratings of Priorities for Improvement. (PDF)

S3 Appendix Histograms of Provider Ratings of Priorities for Improvement. (PDF)

S4 Appendix Additional Priorities Proposed by Providers for Quality Improvement. (PDF)

S5 Appendix Provider Ratings of Priorities for Improvement According to Provider Characteristics. (PDF)

S6 Appendix Selection of Priorities According to Survey Respondent Profession and Potential to Decrease Costs. (PDF)
