# Supplementary material for: Stakeholder Engagement to Identify Priorities for Improving the Quality and Value of Critical Care
Source: PLoS One. 2015 Oct 22;10(10):e0140141. doi: 10.1371/journal.pone.0140141 (PMC4619641; doi:10.1371/journal.pone.0140141)
Supplement: S1 Appendix — (PDF) [file pone.0140141.s001.pdf]

S1 Appendix. Focus Group Guide

- The purpose of this group today is to engage stakeholders to identify patient care practices (overuse, underuse or misuse of care) for reassessment as well as prioritization criteria.
- We know that many of you have been previously involved in prioritization efforts related to critical care. How is this different? How will it build on what has previously been done?
    - Province wide – we will target all ICUs – adult & pediatric in the Province
    - All stakeholder groups –providers, managers & decision-makers
    - Goal is to capture a large number of diverse perspectives – aiming for over a hundred participants
    - Identify prioritization criteria

INTRODUCTIONS and OVERVIEW after above done in group setting (5 min)

- Melissa, Tom (name, position, affiliations, professional roles and responsibilities and interests)
- Each participant (name, where from)
- Explain the purpose of the focus group, what we will do, how the information will be used
- So today we are asking you to reflect on your experiences to suggest those criteria or areas of care. For each topic we discuss, we will give each of you an opportunity to express an opinion, and will then open it up for general discussion. After discussion of each topic we will summarize the key criteria or themes suggested by your opinions and discussion to confirm that we understood correctly

We are interested in identifying patient care practices where there may be important evidence-care gaps. For our purposes we will think of evidence as the best science available to inform best patient practices. An evidence-care gap is when best patient care practices are NOT used. The amount of supporting science is going to vary for different patient care practices. Please think in terms of the current best level of science available recognizing that this could change in the future. We also want to restrict our focus to daily care practices. These don't have to be things we do for every patient, but if we focus on common things then we have a greater chance to have a big impact.

Evidence-Care Gaps (30 min)

Let's talk a little bit about the daily care practices in critical care medicine. From a clinical perspective, what areas of care or specific therapies do you think we **underuse** in the treatment of critically ill patients?

<u>Prompts:</u>

Are there any daily patient care practices that evidence supports the use but are currently underused in daily care practice? Underuse refers to when science shows an intervention to be effective and it is not prescribed. (*Note* – use only to start the discussion if no practices are suggested: For example, there is strong evidence to support prophylaxis to prevent venous thromboembolism (VTE) and yet not all ICU patients receive this best practice. In these circumstances more may be better.)

Let's talk a little bit about the daily care practices in critical care medicine. From a clinical perspective, what areas of care or specific therapies do you think we **overuse** in the treatment of critically ill patients?

<u>Prompts:</u>

Are there any daily patient care practices that evidence supports the use but are currently overused in daily care practice? By overuse I mean when scientific evidence shows an intervention to be ineffective or harmful and it is prescribed. (*Note* – use only to start the discussion if no practices are suggested: For example, there is strong evidence that using intensive insulin therapy in critically ill patients is not helpful and may be harmful and yet it is still prescribed. In these circumstances less may be better than more.)

Let's talk a little bit about the daily care practices in critical care medicine. From a clinical perspective, what areas of care or specific therapies do you think we **misuse** in the treatment of critically ill patients?

<u>Prompts:</u>

How about therapies that you would consider are currently misused? Misuse refers to when scientific evidence shows an intervention to be effective, but it is prescribed for the wrong patients. (*Note* – use only to start the discussion if no practices are suggested: For example, there is strong evidence that albumin (compared to less expensive non-blood product alternatives) is an effective fluid replacement therapy for select patients with liver disease, but not for most ICU

patients. However, many ICU patients are prescribed albumin while many liver disease patients are not. In these circumstances more for some and less for others would be better.

PRIORITIZATION CRITERIA (20 min)

As we noted earlier, one of our goals is to develop a list of criteria that can be used to prioritize evidence-care gaps.

- Review the daily care practices
- Ask the group to identify potential prioritization criteria (e.g., effectiveness, efficiency, safety, patient-centered, equitable, costs, strength of evidence)

QUESTIONS / CONCLUSION (5 min)

Reflecting on our lists of both patient care practices and prioritization criteria, is there anything else we are missing?

Ask if there are any questions about the overall research project, the focus group, what happens next?

Thank them for participating!
