# Supplementary material for: Stakeholder Engagement to Identify Priorities for Improving the Quality and Value of Critical Care
Source: PLoS One. 2015 Oct 22;10(10):e0140141. doi: 10.1371/journal.pone.0140141 (PMC4619641; doi:10.1371/journal.pone.0140141)
Supplement: S2 Appendix — (PDF) [file pone.0140141.s002.pdf]

S2 Appendix. Critical Care Network Core Committee Ratings of Priorities for Improvement

Priorities for Quality Improvement	Median Score on 9 Point Scale	
	Round 1	Round 2
Transition of patient care from ICU to hospital ward	8	8
Early mobilization	8	8
Delirium screening & diagnosis	8	7
Transition of patient care between providers within ICU	8	7
Establishing daily goals for patient care	8	7
End-of-life care	7.5	7
Daily sedation interruption	7.5	7
Duration of empiric antimicrobial prescriptions	7	7
Patient and family participation in daily rounds	7	7
Strategies to facilitate patient day/night cycle	7	7
Temperature control in patients after resuscitation from cardiac arrest	7	7
Routine blood tests	7.5	6.5
Strategies to preserve patient sleep	7	6.5

Physical and pharmacological restraints	7	6.5
Matching available human resources to patient needs	7.5	6
Limiting blood transfusion in non-bleeding patients	7	6
Prescription of empiric broad spectrum antimicrobials	7	6
Establishing goals of care at time of patient admission to ICU	7	6
Reviewing goals of care at time of patient discharge from ICU	7	6
Routine diagnostic imaging studies	8	5.5
Venous thromboembolism prophylaxis	7.5	5
Placing patients with acute respiratory distress syndrome in prone position	7	5
Fluid resuscitation with albumin	7	5
Protocols to guide liberation from mechanical ventilation	7	5
Patient care rounds that are multidisciplinary	7	5
Frequency of communication with patient/family	7	5
Lung protective ventilation strategies for patients with acute respiratory distress syndrome	6	5
Bronchoscopy to evaluate ventilator associated pneumonia	6	5

Request for tissue/organ donation from families of deceased patients	6	5
Skin hygiene to prevent pressure ulcers	6	5
Post-operative monitoring of elective surgical patients	6	5
Facilitating full scope of practice for all ICU providers	5.5	5
Providing enteral nutrition	5	5
Precautionary isolation of patients pending screening results for multidrug resistant organisms	5	5
Prescriptions of inhaled Nitric Oxide for hypoxemic respiratory failure	8	4.5
Reporting adverse drug events	7.5	4.5
Medication reconciliation	7	4.5
Ventilation with high frequency oscillation for patients with acute respiratory distress syndrome	7	4.5
Using ultrasound to guide central venous catheter insertion	7	4.5
Strategies to prevent ventilator associated pneumonia	7	4.5
Serial CT scans of the head for brain injured patients	6	4.5
Non-invasive hemodynamic monitoring	5.5	4.5
Stepping medications from intravenous to oral route	7	4

Duration of central venous catheter	6.5	4
Patient care rounds that are standardized in structure & process	6	4
Monitoring patient radiation exposure from diagnostic studies/procedures	6	4
Type of dialysis prescribed (i.e., continuous renal replacement therapy vs. intermittent renal replacement therapy)	5	4
Oral hygiene (e.g., brushing teeth)	7	3.5
Frequency of central venous catheter insertion	5.5	3.5
Protocols to manage sepsis	7	3
Stress ulcer prophylaxis	6	3
Routine prescription of bronchodilators for patients mechanically ventilated	6	3
CT scans of the head for patients "not waking up"	5	3
Chest physiotherapy for patients with respiratory failure	5	3
Frequency of invasive hemodynamic monitoring (i.e. arterial catheters)	5	3
Using insulin to provide tight glycemic control	5	3
Non-invasive ventilation for patients with respiratory failure	5	3
Providing total parenteral nutrition	5	3

Patient hygiene (e.g., bathing)	6.5	2
Selective decontamination of the digestive tract to prevent sepsis	5	2
Frequency of therapeutic drug monitoring (e.g., phenytoin levels)	5	2
Duration of prescriptions for progastric motility medications	5	2
Surveillance blood cultures	5	2
Requests for subspecialty consultation	5	2
Flushing invasive catheters with heparin	5	2
Fluid resuscitation with hydroxyethyl starch solutions	5.5	1
Prescribing acetaminophen using weight-based doses	5	1
Modified barium swallow to assess swallowing post-extubation	3	1
