# Supplementary figures and images for: Stakeholder Engagement to Identify Priorities for Improving the Quality and Value of Critical Care
Source: PLoS One. 2015 Oct 22;10(10):e0140141. doi: 10.1371/journal.pone.0140141 (PMC4619641; doi:10.1371/journal.pone.0140141)

S3 Appendix. Histograms of Provider Ratings of Priorities for Improvement

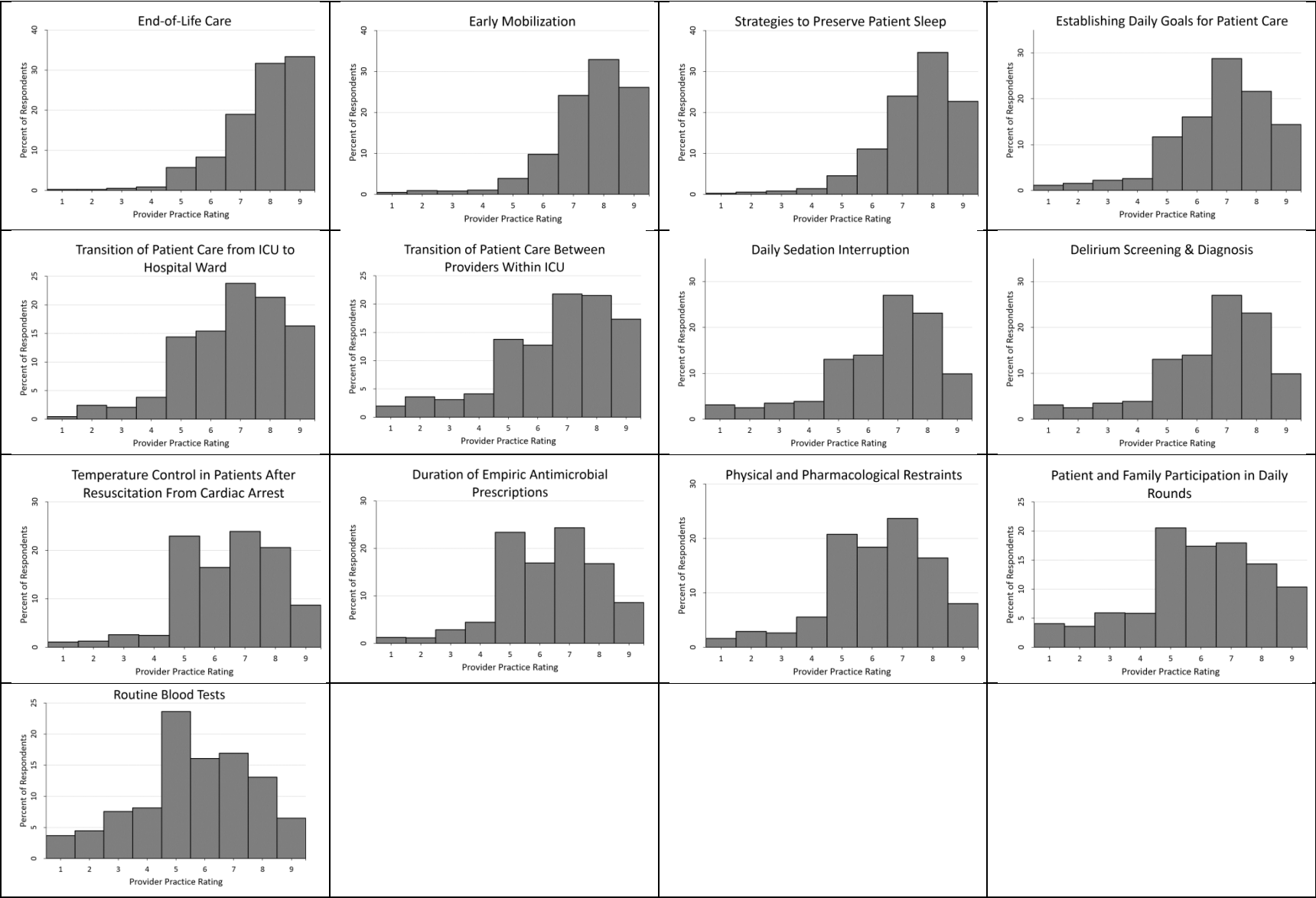

Supplement: S3 Appendix — (PDF) [file pone.0140141.s003.pdf]
