# Supplementary material for: Stakeholder Engagement to Identify Priorities for Improving the Quality and Value of Critical Care
Source: PLoS One. 2015 Oct 22;10(10):e0140141. doi: 10.1371/journal.pone.0140141 (PMC4619641; doi:10.1371/journal.pone.0140141)
Supplement: S4 Appendix — (PDF) [file pone.0140141.s004.pdf]

S4 Appendix. Additional Priorities Proposed by Providers for Quality Improvement*

Priorities Proposed by Providers	Providers (n=136)
Newly Identified Priorities	
1. Transition of patient care into ICU	9
2. Infection control	6
3. Family access to support staff	5
4. Allowing family to be with patient as needed	3
5. Bowel management	2
6. Informing patients of post-ICU syndrome	2
7. Delirium management	1
8. Family presence during emergency procedures	1
9. Fluid management	1
10. Inviting family to be part of care team	1
11. Keeping patient information private	1
12. Management of thirst in ventilated patients	1
13. Patient/family-care team introduction	1
14. Patient transport	1
15. Patient's inability to communicate	1
16. Post-operative care	1
17. Sedation and analgesia	1
18. Timely updates for major changes in patient status	1
19. Use of invasive procedures	1

Priorities Considered but not Selected by Network Committee

1. Establishing goals of care at time of patient admission to ICU	12
2. Multidisciplinary rounds	7
3. Providing enteral nutrition	7
4. Duration of central venous catheter	4
5. Medication reconciliation	4
6. Oral hygiene (e.g., brushing teeth)	3
7. Protocols to guide liberation from mechanical ventilation	3
8. Skin hygiene to prevent pressure ulcers	3
9. Venous thromboembolism prophylaxis	3
10. Chest physiotherapy for patients with respiratory failure	2
11. Frequency of communication with patient/family	2
12. Limiting blood transfusion in non-bleeding patients	2
13. Non-invasive hemodynamic monitoring	2
14. Request for tissue/organ donation for families of deceased patients	2
15. Routine diagnostic imaging studies	2
16. Using insulin to provide tight glycemic control	2
17. Strategies to prevent ventilator associated pneumonia	2
18. Bronchoscopy to evaluate patients with ventilator associated pneumonia	1
19. Fluid resuscitation with albumin	1
20. Fluid resuscitation with hydroxyethyl starch solutions	1
21. Frequency of invasive hemodynamic monitoring (i.e., arterial	1

catheters)	
22. Lung protective ventilation strategies for patients with acute respiratory distress syndrome	1
23. Matching available human resources to patient needs	1
24. Placing patients with acute respiratory distress syndrome in prone position	1
25. Protocols to manage sepsis	1
26. Stress ulcer prophylaxis	1
27. Type of dialysis prescribed (i.e., continuous renal replacement therapy vs. intermittent renal replacement therapy)	1
28. Using ultrasound to guide central venous catheter insertion	1
<u>Priorities Considered and Selected by Network Committee</u>	
1. Transition of patient care between providers within ICU	8
2. Transition of patient from ICU to hospital ward	4
3. Physical and pharmacological restraints	2
4. Daily sedation interruption	1
5. Early mobilization	1
6. End-of-life care	1
7. Prescriptions of empiric broad spectrum antimicrobials	1
8. Strategies to preserve patient sleep	1

* 136 providers suggested an additional 175 priorities. 43 suggestions did not satisfy the inclusion for the study. Among those suggestions that did satisfy the study inclusion criteria, a total 55 unique priorities were identified, 19 of which were newly identified, 28 had been considered but not selected by the Network committee and 8 had been considered and selected by the Network committee.
