# Supplementary material for: Stakeholder Engagement to Identify Priorities for Improving the Quality and Value of Critical Care
Source: PLoS One. 2015 Oct 22;10(10):e0140141. doi: 10.1371/journal.pone.0140141 (PMC4619641; doi:10.1371/journal.pone.0140141)
Supplement: S5 Appendix — (PDF) [file pone.0140141.s005.pdf]

S5 Appendix. Provider Ratings of Priorities for Improvement According to Provider Characteristics*'†

	Profession				Provider Experience			Institution Academic Status		Institution Location		Patient Population	
**Practice**	**MD**	**RN**	**RT**	**Allied Health**	**<10 years**	**10-20 years**	**>20 years**	**Teaching**	**Non-teaching**	**Urban**	**Regional**	**Adult**	**Pediatric**
End-of-life care	8	8	8	8	8	8	8	8	8	8	8	8	8
Early mobilization	8	8	8	8	8	8	8	8	8	8	8	8	7
Strategies to preserve patient sleep	7	8	8	7	8	8	8	8	8	8	8	8	7
Establishing daily goals for patient care	7	7	7	7	7	7	7	7	7	7	7	7	7
Transition of patient care from ICU to hospital ward	7	7	7	8	7	7	8	7	7	7	7	7	7
Transition of patient care between providers within ICU	7	7	7	7.5	7	7	8	7	7	7	7	7	7
Daily sedation interruption	7	7	7	8	7	7	7	7	7	7	8	7	5.5
Delirium screening & diagnosis	7	7	7	8	7	7	8	7	7	7	7	7	6
Temperature control in patients after resuscitation from cardiac arrest	6	7	7	7	7	7	7	7	7	7	7	7	7

Duration of empiric antimicrobial prescriptions	7	6	6	7	6	6	7	7	6	6	7	6	7
Physical and pharmacological restraints	5.5	7	7	6	6	6	7	6	7	6	6	6	7
Patient and family participation in daily rounds	6	6	6	7	6	6	7	6	6	6	6	6	7
Routine blood tests	6	6	5	5	6	6	6	6	6	6	5	6	6

* Data presented as medians. Shaded cells represent priorities rated as necessary or supplementary by different provider groups

† Abbreviations: MD, physician; RN, nurse; RT, respiratory therapist
