# Supplementary material for: Stakeholder Engagement to Identify Priorities for Improving the Quality and Value of Critical Care
Source: PLoS One. 2015 Oct 22;10(10):e0140141. doi: 10.1371/journal.pone.0140141 (PMC4619641; doi:10.1371/journal.pone.0140141)
Supplement: S6 Appendix — (PDF) [file pone.0140141.s006.pdf]

S6 Appendix. Selection of Priorities According to Survey Respondent Profession and Potential to Decrease Costs

Provider Profession	Adjusted Odds Ratio (95% confidence interval)*		P-value for Interaction Term*
	Unknown or No Potential to Decrease Costs	Potential to Decrease Costs	
			<0.001
Physician†	1.0	1.0	
Nurse	1.56 (1.15-2.13)	0.83 (0.63-1.09)	
Respiratory Therapist	1.69 (1.20-2.39)	0.81 (0.59-1.10)	
Allied Health	2.37 (1.63-3.46)	1.19 (0.85-1.67)	

* Odds ratio and p-values adjusted for provider (profession, years of experience in critical care, academic status of ICU) and patient care practice characteristics (strength of supporting evidence, potential to benefit patient, potential to improve patient/family experience, potential to decrease costs, ability to easily measure the practice, ability to take action to change the practice) using random effects logistic regression model.

† Providers with this factor served as the reference group.
